# T360Fusion: Temporal 360 Multimodal Fusion for 3D Object Detection via Transformers

**DOI:** 10.3390/s25164902

**Published:** 2025-08-08

**Authors:** Khanh Bao Tran, Alexander Carballo, Kazuya Takeda

**Affiliations:** 1 Graduate School of Informatics, Nagoya University, Furo-cho, Chikusa-ku, Nagoya 464-8601, Japan; takeda@g.sp.m.is.nagoya-u.ac.jp; 2 Faculty of Engineering and Graduate School of Engineering, Gifu University, 1-1 Yanagido, Gifu 501-1193, Japan; alex@gifu-u.ac.jp; 3 Institutes of Innovation for Future Society, Nagoya University, Furo-cho, Chikusa-ku, Nagoya 464-8601, Japan; 4 Tier IV Inc., Nagoya University Open Innovation Center, 1-3, Meieki 1-chome, Nakamura-cho, Nagoya 450-6610, Japan

**Keywords:** LiDAR sensors, 360 RGB cameras, 360 thermal cameras, feature-level fusion, object detection, transformers

## Abstract

Object detection plays a significant role in various industrial and scientific domains, particularly in autonomous driving. It enables vehicles to detect surrounding objects, construct spatial maps, and facilitate safe navigation. To accomplish these tasks, a variety of sensors have been employed, including LiDAR, radar, RGB cameras, and ultrasonic sensors. Among these, LiDAR and RGB cameras are frequently utilized due to their advantages. RGB cameras offer high-resolution images with rich color and texture information but tend to underperform in low light or adverse weather conditions. In contrast, LiDAR provides precise 3D geometric data irrespective of lighting conditions, although it lacks the high spatial resolution of cameras. Recently, thermal cameras have gained significant attention in both standalone applications and in combination with RGB cameras. They offer strong perception capabilities under low-visibility conditions or adverse weather conditions. Multimodal sensor fusion effectively overcomes individual sensor limitations. In this paper, we propose a novel multimodal fusion method that integrates LiDAR, a 360 RGB camera, and a 360 thermal camera to fully leverage the strengths of each modality. Our method employs a feature-level fusion strategy that temporally accumulates and synchronizes multiple LiDAR frames. This design not only improves the detection accuracy but also enhances the spatial coverage and robustness. The use of 360 images significantly reduces blind spots and provides comprehensive environmental awareness, which is especially beneficial in complex or dynamic scenes.

## 1. Introduction

Perception systems for autonomous vehicles and robotic platforms have made rapid progress in recent years. Among the many emerging approaches, multimodal sensor fusion has become a prominent research direction [1,2], enabling systems to take advantage of the complementary strengths of various sensor modalities. RGB cameras offer detailed color and texture information, which is valuable for object classification under well-lit conditions. Thermal cameras capture heat signatures, making them effective in low light or visually obscured environments. Meanwhile, LiDAR sensors generate dense and accurate 3D point clouds, supporting precise spatial location and geometric reasoning regardless of ambient lighting. However, each sensor type also has inherent limitations [3,4]. RGB cameras are highly sensitive to illumination and may fail in darkness or in adverse weather. Thermal cameras often suffer from low spatial resolution and reduced effectiveness when temperature contrasts are weak. LiDAR sensors, though accurate, can struggle with certain material surfaces and exhibit sparsity at longer distances. These limitations have motivated the development of sensor fusion techniques to construct more robust and comprehensive perception systems [5,6,7,8,9,10]. Fusion methods are generally categorized into three types, which are early fusion, intermediate fusion, and late fusion. Late fusion combines outputs at the decision level, offering simplicity. In recent years, the approach of combining multiple fusion strategies has attracted attention. Early fusion integrates raw sensor data at the input level, leading to higher accuracy. Intermediate fusion balances accuracy and computational speed by fusing intermediate feature representations.

To enhance the effectiveness of point cloud acquisition, we employ multiple LiDAR sensors with complementary characteristics, leveraging the specific strengths of each type [2,4]. While some LiDARs offer superior stability and performance in close-range environments, others are optimized for long-range sensing. Combining different LiDAR types enables the system to compensate for individual sensor limitations and helps mitigate the density imbalance of point clouds, ensuring high-resolution perception across both near and far ranges. However, deploying multiple LiDAR sensors in close proximity introduces the risk of sensor interference, where overlapping laser pulses may degrade measurement quality. These practical challenges underscore the need for a robust calibration strategy that not only ensures accurate spatial and temporal alignment, but also explicitly models the unique characteristics of each sensor to achieve coherent and reliable multi-LiDAR fusion. In addition, the integration of LiDAR with 360-degree RGB and thermal cameras represents a significant advancement over conventional narrow field-of-view systems. Panoramic imaging mitigates blind spots, providing omnidirectional perception ideal for complex urban scenarios [11,12,13]. Motivated by these benefits, we propose a temporally aware feature-level fusion approach that accumulates sequential LiDAR frames and aligns them with 360-degree images for robust multimodal perception.

**Figure 1:**
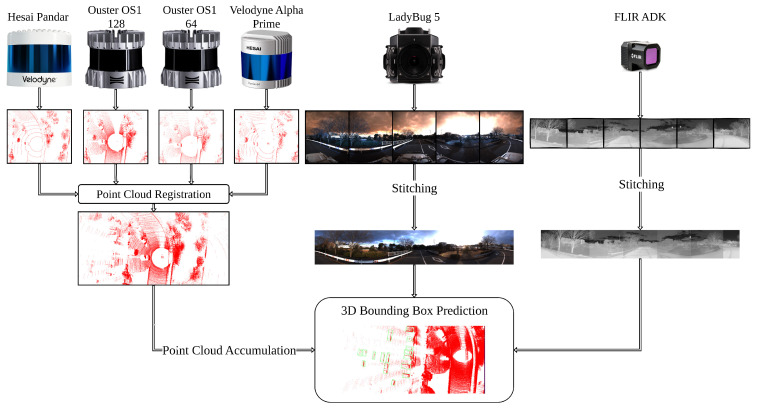
Visualization of the system including RGB cameras, thermal cameras, Ouster OS1-128, Ouster OS1-64, Velodyne Alpha Prime, and Hesai Pandar. A 360 RGB camera is stitched from five RGB cameras and a 360 thermal camera is stitched from six cameras. Point cloud data from all four LiDAR sensors are accumulated to form a dense and comprehensive 3D representation of the environment. The multimodal data are then fused within the detection pipeline to perform robust object detection.

Figure 1 illustrates the overall architecture of the proposed system. Four LiDAR sensors are first calibrated to form a unified 3D acquisition system, which significantly increases the spatial coverage and density of point cloud data. In addition to that, a set of RGB cameras and a set of thermal cameras are stitched to construct two 360 cameras, respectively. This 360 design ensures full environmental awareness from both visual and thermal perspectives. To further enhance spatial resolution and compensate for temporal sparsity or occlusions, multiple consecutive LiDAR frames are temporally accumulated. This accumulation enhances point density, preserving critical geometry despite occlusions or material properties. The resulting fused LiDAR frames are then aligned with the 360 RGB and thermal images through geometric calibration. Finally, the aligned multimodal data are passed through a unified early-fusion and middle-fusion framework for object detection. This architecture allows the system to learn in two stages, contributing to robust and accurate object recognition. The main contributions of this work are summarized as follows:

1. We propose a novel calibration strategy specifically designed to enhance alignment accuracy between multiple LiDAR sensors. By addressing both spatial discrepancies and temporal misalignments, this strategy improves global consistency in point cloud integration. It is particularly effective in setups involving wide fields of view or overlapping sensor coverage, ensuring that the fused point clouds maintain geometric fidelity and can be reliably used in object detection.
2. We developed an enhanced multimodal fusion framework that jointly utilizes LiDAR point clouds, 360-degree RGB images, and 360-degree thermal images to achieve robust object detection in complex environments. Each modality contributes complementary information. LiDAR point clouds provide precise spatial geometry, RGB images capture rich texture and color cues, and thermal images ensure reliability under poor lighting or adverse weather. Our fusion approach is designed to fully exploit 360-degree perception while maintaining spatial and temporal consistency across the modalities. As a result, the system is resistant to sensor-specific limitations that can affect single-modality detection.
3. Our implementation, including code and dataset, will be made publicly available at https://github.com/baokhanhtran/T360Fusion, accessed on 26 July 2025.

This paper is structured as follows. Section 2 provides a detailed overview of the state of the art in sensor fusion and calibration techniques, with a particular emphasis on approaches relevant to LiDAR and camera systems. This section serves as the foundation for understanding the technical motivations behind our proposed methods. Section 3 introduces our novel calibration strategy, which addresses both LiDAR-to-LiDAR and LiDAR-to-camera alignment. We describe the methodology in detail and highlight its adaptability in dynamic and targetless settings. Section 4 outlines the architecture and design of our multimodal fusion framework for 3D object detection, emphasizing the integration of LiDAR point clouds, 360-degree RGB images, and thermal imaging. Section 5 presents a detailed quantitative and qualitative evaluation of the proposed methods, including ablation studies and performance benchmarks. Section 6 offers in-depth discussions on the implications of our findings, potential limitations, and directions for future research. Finally, Section 7 concludes the paper by summarizing our key contributions and highlighting the broader impact of our work on the field of autonomous driving and multimodal perception.

## 2. Related Works

In this section, we will provide a summary of the main issues addressed in this paper, including the calibration method and the fusion techniques for object detection. The calibration method discussed is a targetless calibration. This technique is used to calibrate the sensors without predefined reference points. For object detection, we will use early and intermediate fusion for thermal and RGB images to enhance the performance of LiDAR sensors. This is an approach that can optimize the use of the advantageous characteristics of each type of sensor, thereby improving the detection efficiency.

### 2.1. Target Calibration

The most widely adopted approach for LiDAR and camera calibration is the target-based method, which involves placing known geometric patterns or calibration targets in fixed locations within the environment [14,15]. These targets serve as reference structures that are simultaneously visible across different sensor modalities, allowing the extraction of correspondence features. By capturing the same target from multiple viewpoints and distances, the system can estimate the parameters of each sensor [16]. Among the various types of calibration targets, black and white checkerboards are the most commonly used, as in the Autoware package [17] because of their simple design and strong visual contrast, which facilitates reliable corner detection in RGB images. The choice of target material and shape is often tailored to the specific characteristics of each sensor type [18].

**Figure 2:**
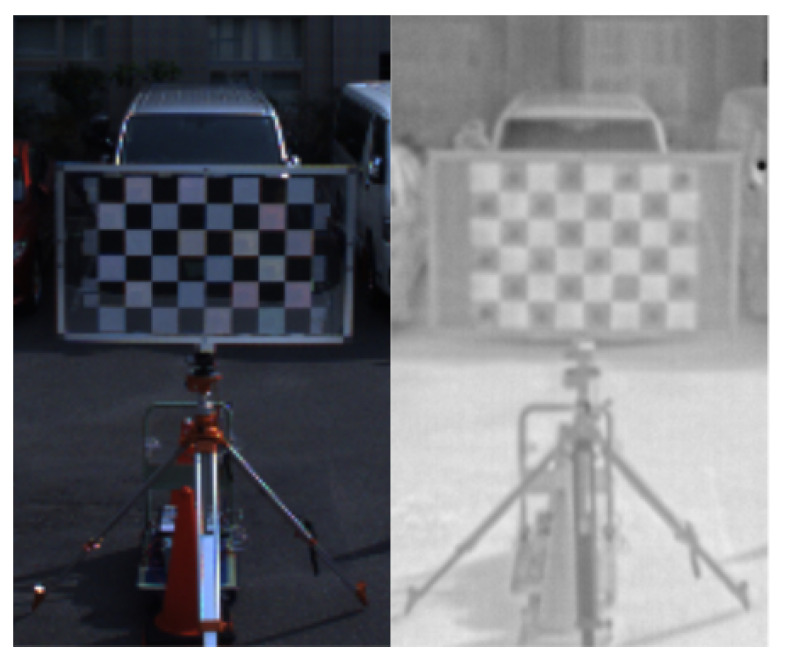
Visualization of the checkerboard scanned by two types of cameras. The **left image** shows the data from the RGB camera and the **right image** shows the data from the thermal camera.

In this paper, to support the unique requirements of 360-degree RGB and 360-degree thermal cameras, we introduce a custom-designed calibration target, as illustrated in Figure 2 [19]. The front side of the target employs a standard black and white checkerboard to enable feature detection in RGB images. The back side integrates a temperature-controlled system to assist thermal cameras in recognizing the pattern. This enables accurate calibration between RGB and thermal modalities, even under varying environmental lighting or when relying solely on thermal data. The dual-sided design ensures that both types of panoramic camera can observe the same spatial references from multiple angles simultaneously, thereby facilitating accurate and robust sensor alignment. We use the Autoware package for camera calibration.

### 2.2. Targetless Camera–LiDAR Calibration

This method enables sensor calibration to be performed without the need for dedicated calibration targets or physical markers [20,21]. Rather than relying on predefined patterns, such as checkerboards or specially designed calibration boards, the approach takes advantage of naturally occurring features in the environment to estimate the necessary transformation parameters. This targetless calibration method increases flexibility and practicality, particularly in dynamic or unstructured environments where setting up physical targets may be infeasible. In this study, we adopt a feature-based calibration technique that operates on multimodal sensor data captured from LiDAR, RGB cameras, and thermal cameras, all with overlapping fields of view. The method identifies and extracts meaningful geometric features from both the 3D point clouds generated by the LiDAR and the 2D images obtained from the cameras. Once the features are extracted, the calibration parameters are estimated by optimizing a cost function that minimizes the spatial discrepancy between the LiDAR points projected into the image space and their corresponding visual features detected in the camera images.

### 2.3. Targetless Multi-LiDAR Calibration

Calibration between multiple LiDAR sensors is an important task in autonomous systems, especially for applications that require dense, wide-field 3D perception. The goal is to determine the extrinsic transformation between each LiDAR coordinate system, allowing an accurate fusion of point clouds [22,23,24]. To overcome the limitations of target-based methods, recent research has turned to target-free or environment-based calibration approaches that estimate sensor alignment using natural scene structure. Current approaches can be broadly categorized into two groups: learning-based methods and geometrically constrained methods. Learning-based techniques or probabilistic frameworks offer increased robustness in noisy, dynamic, or unstructured environments by learning patterns of spatial correlation or motion from data. These approaches can generalize well, but often require extensive training data and careful network design to avoid overfitting. In contrast, geometrically constrained methods rely directly on the spatial consistency of features observed by multiple sensors. For example, alignment can be estimated by minimizing the discrepancy between corresponding geometric primitives, such as planes, edges, or surface normals in LiDAR point clouds.

### 2.4. Early Fusion Strategy

Early fusion is one of the fundamental approaches widely employed in multimodal object detection tasks. This method involves the direct integration of raw data from multiple sensor modalities at the input [5,25,26]. By fusing sensor data at this level, early fusion aims to capture the inherent correlations and complementary information between different methodologies. For example, RGB images provide rich color and texture, LiDAR delivers precise intensity or depth, and thermal images offer strong visibility under poor lighting conditions. The fusion of raw data improves robustness and contextual understanding in complex environments. One of the key advantages of early fusion lies in its computational efficiency. Early fusion facilitates more comprehensive spatial and semantic representations, as the network can leverage multimodal signals simultaneously during training and inference, potentially leading to higher object detection accuracy. However, early fusion has several challenges, most notably the need for precise temporal and spatial synchronization across sensor data streams. Disparities in resolution and frame rates require careful pre-processing and alignment to ensure consistent data representation. Moreover, integrating heterogeneous raw data formats into a unified input space often requires complex transformation mechanisms, which can increase implementation complexity.

### 2.5. Intermediate Fusion Strategy

Intermediate fusion is another widely adopted strategy in multimodal object detection, where data from different sensor modalities are processed independently through initial layers and then fused at an intermediate feature level [6,9,10,27,28]. Rather than combining raw input directly, this method extracts modality-specific features before integrating them, allowing each sensor to retain its unique characteristics while still contributing to a unified representation. These features are then aligned and fused to form a joint representation that captures cross-modal relationships with a higher-level semantic context. A primary advantage of intermediate fusion lies in its flexibility because this approach allows the system to take advantage of the full potential of each sensor without being constrained by discrepancies in raw data formats or resolution. Moreover, fusion at the feature level offers a balance between the richness of early fusion and the simplicity of late fusion, often leading to improved detection accuracy and better generalization. However, intermediate fusion also presents specific challenges. Aligning and merging intermediate features from heterogeneous networks requires careful architectural design, especially when feature dimensions and spatial resolutions differ. Additionally, increased model complexity and memory requirements affect scalability and performance.

## 3. Multimodal Calibration

To enhance spatial coverage and reduce the occurrence of missing points caused by occlusions, material reflectivity, or limited sensor field of view, we employ a multi-LiDAR setup consisting of four different LiDAR sensors. Each LiDAR offers distinct advantages in resolution and vertical coverage. By strategically combining data from these complementary sensors, we effectively compensate for blind spots or sparse regions that would otherwise remain unobserved when relying on a single LiDAR. This configuration enables robust 3D scene reconstruction in complex urban environments [29].

**Figure 3:**
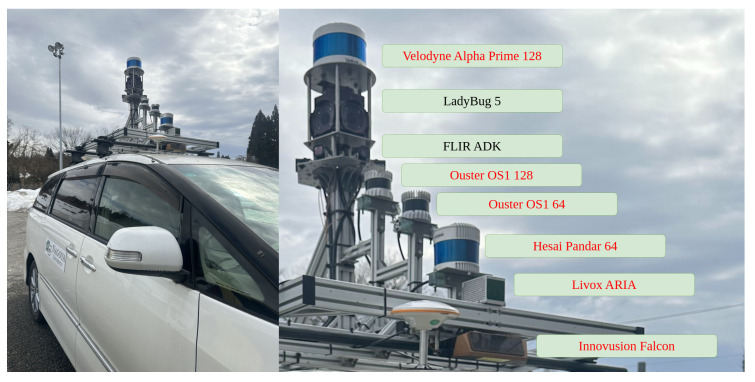
Our system includes the following sensors: LiDAR Velodyne Alpha Prime, LadyBug-5 camera, 6 FLIR ADK cameras, LiDAR Ouster-128, LiDAR Ouster-64, and LiDAR Hesai Pandar.

**Figure 4:**
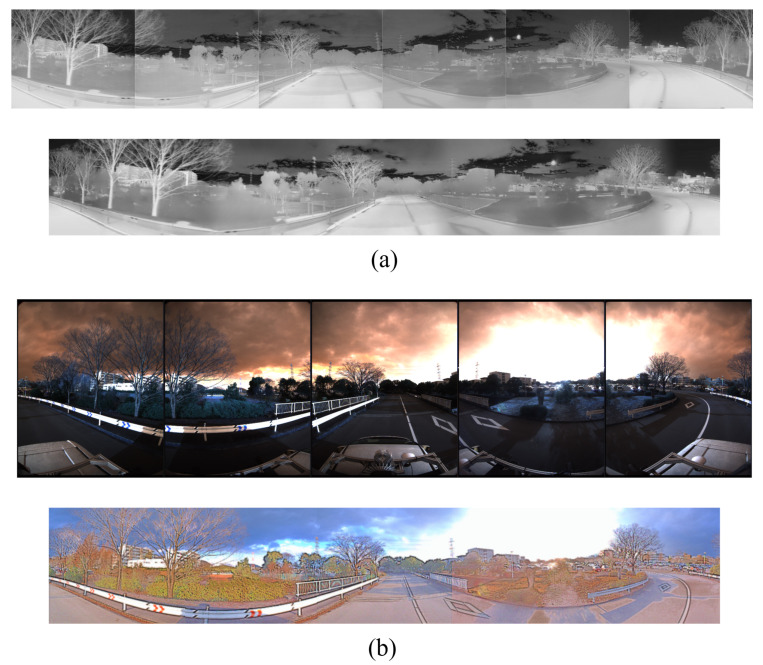
Visualization of stitching (**a**) 360 thermal images and (**b**) 360 RGB images.

In this paper, we used Ouster-128 LiDAR, Ouster-64 LiDAR, Velodyne Alpha Prime LiDAR, Hesai Pandar LiDAR [30], six FLIR ADK cameras [31], and the LadyBug 5 camera [32] as shown in Figure 3 to record the dataset and evaluate the results. LadyBug camera and the FLIR ADK cameras were arranged in a unified structure to capture 360-degree images. The 360 RGB camera and the 360 thermal camera were combined, as shown in Figure 4. For RGB images, we applied the Retinex algorithm to enhance color fidelity and improve visibility under varying lighting conditions. For thermal images, contrast-limited adaptive histogram equalization was employed to normalize information across the six thermal cameras, enhancing local contrast and preserving structural details.

**Figure 5:**
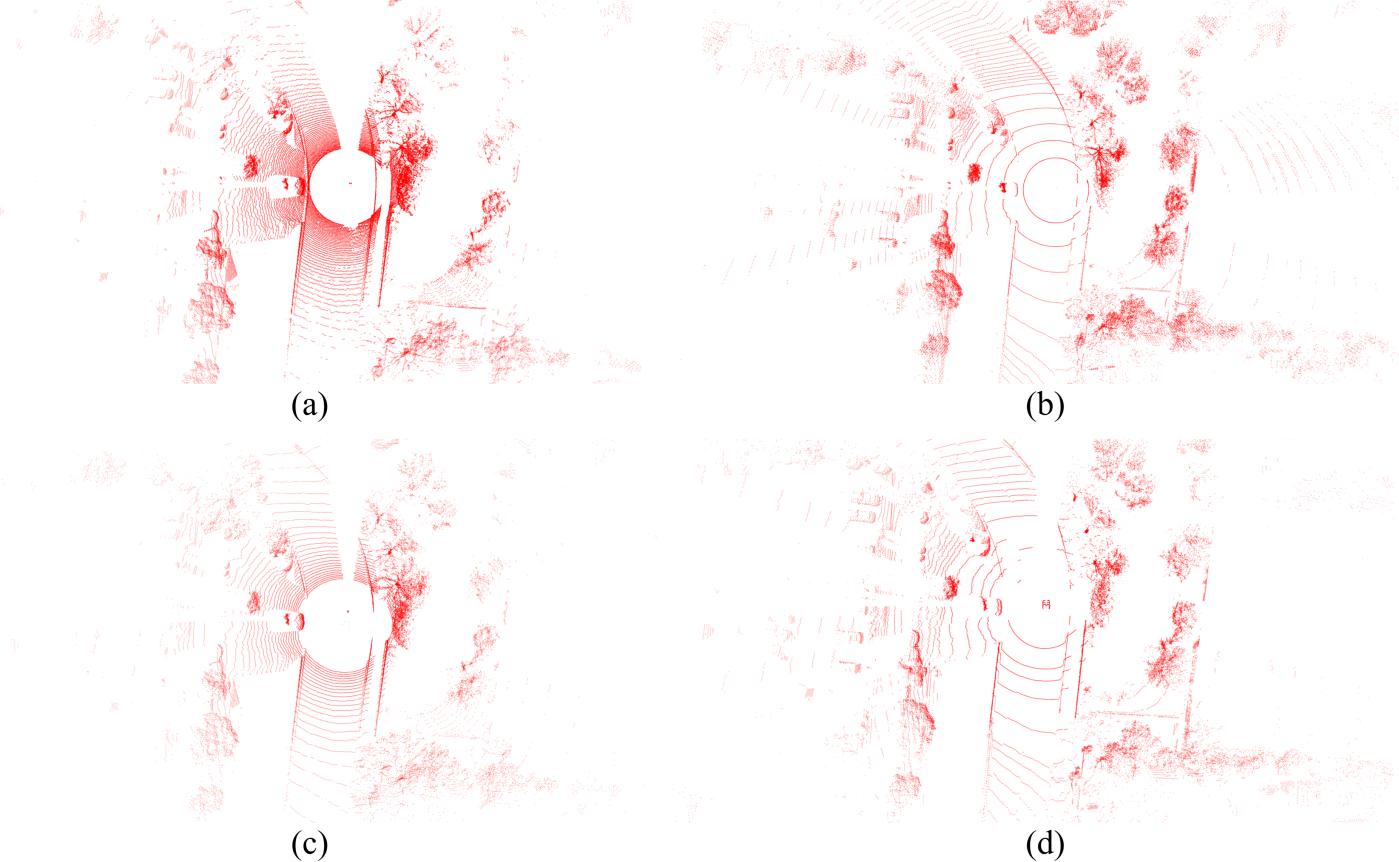
Visualization of the scanning results of four types of LiDAR sensors. (**a**) The data from Ouster-128 LiDAR. (**b**) The data from Velodyne Alpha Prime. (**c**) The data from Ouster-64. (**d**) The data from Hesai Pandar.

Figure 5 shows the result of projecting the point clouds of four LiDAR sensors into the 3D space. The Ouster OS1-128 LiDAR captures data through 128 vertical channels, providing exceptionally dense point clouds at close range. This high density is particularly effective for capturing fine-grained geometric details of objects at close or mid-distance, making it well suited for extracting structural features and precise object boundaries. However, one limitation of this sensor is its sensitivity to material and surface properties; objects with low reflectivity, unusual colors, or complex shapes may result in a lack of or missing points. The Velodyne Alpha Prime also features 128 channels, but is optimized for long-range sensing. Its laser arrangement provides superior point coverage at extended distances, which is critical for early detection and tracking of far-field objects in autonomous driving. However, the point density of this LiDAR is quite low in close proximity, which can introduce difficulties in detecting moving objects near the vehicle.

The Ouster OS1-64 is structurally similar to OS1-128, with a focus on high-density point capture in the close or middle range. Although it produces fewer points overall due to fewer channels, OS1-64 benefits from greater stability and lower data throughput, making it more robust in continuous operation. This sensor effectively complements OS1-128 by filling in the gaps when the latter suffers from point dropouts or signal loss. The Hesai Pandar LiDAR offers a distinct advantage in mid-range and long-range detection. Moreover, the Hesai Pandar is known for its high stability and consistent performance in prolonged operation.

**Figure 6:**
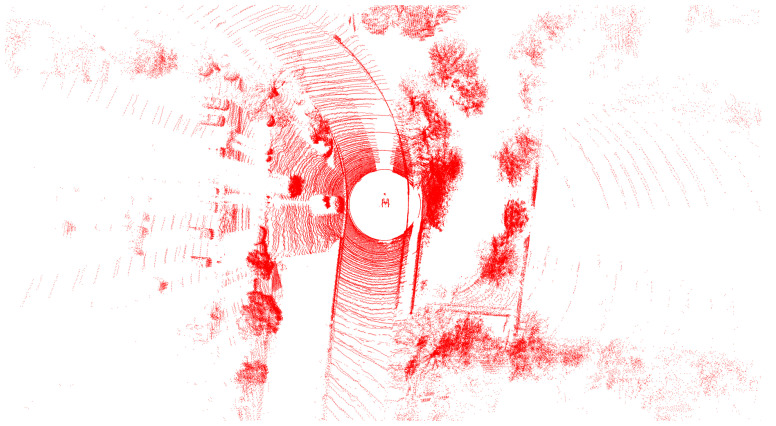
Visualization of accumulated point clouds from four LiDAR sensors.

By integrating these four LiDAR sensors, the system constructs a synthetic perception layer that significantly increases the density of spatial points and the robustness in all distance ranges, as shown in Figure 6. This multi-LiDAR configuration allows the system to generate rich and continuous 3D point clouds within a single frame, capturing detailed geometry from the near field to far field. As a result, the proposed system not only provides full 360-degree coverage but also adapts effectively to the requirements of diverse operational scenarios ranging from urban navigation to high-speed highway driving. In multi-LiDAR fusion scenarios, point cloud alignment across sensors with heterogeneous characteristics is essential for reliable 3D perception.

Iterative Closest Point (ICP) algorithms have been widely adopted as a fundamental solution for rigid registration of 3D point clouds, particularly in LiDAR-based perception systems. The ICP algorithm [33] is known for its conceptual simplicity, ease of implementation, and rapid convergence when the initial misalignment is small. However, its reliance on strict point-to-point correspondence makes it highly sensitive to outliers and sensor noise. To improve robustness, GICP extends the classical formulation by modeling each point as a Gaussian distribution and incorporating local surface geometry through covariance estimation [34]. By blending point-to-point and point-to-plane metrics, GICP enhances convergence in partially overlapping or noisy scenes. However, it remains sensitive to inaccurate normal estimation and introduces considerable computational overhead, particularly when operating on dense point clouds. Several other variants have emerged to address the limitations of classical ICP. VICP improves robustness in dynamic environments but introduces a dependence on the reliability of external motion cues. Beyond these, learning-based methods such as PRNet and RPM-Net leverage deep neural networks to learn feature descriptors and soft correspondences, achieving superior robustness in complex environments. However, these methods require substantial training data and are often less interpretable or generalizable compared to their classical counterparts.

To address the limitations of classical ICP, we propose an improved registration method using the Log-Cosh loss function in the Lie group SE(3) [35]. The Log-Cosh loss function is resistant to outliers typically caused by sensor noise and calibration errors. The Log-Cosh loss function also maintains gradient stability, leading to improved convergence behavior. Furthermore, rather than linearizing the transformation in Euclidean space, we perform optimization directly on SE(3), allowing for more accurate and consistent pose updates. The proposed geometric formulation not only accelerates convergence but also achieves a more effective balance between robustness, accuracy, and computational efficiency. Overall, our method significantly enhances the stability and performance of multi-LiDAR registration, particularly under challenging scenarios such as noisy, sparse, or incomplete point cloud data. However, this gain in accuracy comes at the cost of increased per-iteration computational complexity. As a result, when implemented on the same hardware, the overall runtime may not differ significantly from that of traditional methods, despite the improved convergence results. Overall, our approach significantly enhances the stability and performance of multi-LiDAR registration, especially under challenging conditions such as noisy, sparse, or incomplete point cloud data.

Let $P=\{p_{i}\in R^{3}\}$ be the source point clouds and $Q=\{q_{i}\in R^{3}\}$ be the target point clouds. The goal is to estimate a rigid transformation T∈SE(3) as in Equation (1).

(1) $${\tilde{q}}_{i}=T{\tilde{p}}_{i}=\left[\begin{array}{cc} R & t\\ 0^{⊤} & 1 \end{array}\right]{\tilde{p}}_{i}\;\mathrm{where}\;R\in SO\left(3\right),t\in R^{3},{\tilde{p}}_{i}=\left[\begin{array}{c} p_{i}\\ 1 \end{array}\right],{\tilde{q}}_{i}=\left[\begin{array}{c} q_{i}\\ 1 \end{array}\right]$$

The residual vector for each pair of points is defined as in Equation (2):

(2) $$r_{i}=Rp_{i}+t-q_{i}$$

Using this residual, Equations (3)–(5) together define the Log-Cosh ICP optimization framework, where the classical squared loss function is replaced by the Log-Cosh function, and the transformation is updated iteratively.

(3) $$T^{*}=\arg\min_{T\in SE(3)}\sum_{i=1}^{N}\log\;\cosh\left(∥r_{i}∥\right)$$

After each iteration, T is updated via

(4) $$T\leftarrow \exp\left(\hat{\delta \xi }\right)\cdot T$$

(5) $$\begin{array}{c} \left(\sum_{i=1}^{N}\frac{\tanh\left(∥r_{i}∥\right)}{∥r_{i}∥}\;J_{i}^{⊤}J_{i}\right)\delta \xi =-\sum_{i=1}^{N}\left(\frac{\tanh\left(∥r_{i}∥\right)}{∥r_{i}∥}\;J_{i}^{⊤}r_{i}\right)\;\mathrm{where}\;J_{i}=\frac{\partial r_{i}}{\partial [t,\theta ]} \end{array}$$

In this study, we build on our previous research [36,37]. These studies contribute to a feature-based, targetless calibration framework for multimodal sensor systems. Unlike conventional target-based approaches, which rely on artificial markers or controlled environments, the targetless paradigm demonstrates superior adaptability to unstructured and dynamic scenes, eliminates the logistical overhead of target deployment, and enhances generalizability across varying sensor configurations.

**Figure 7:**
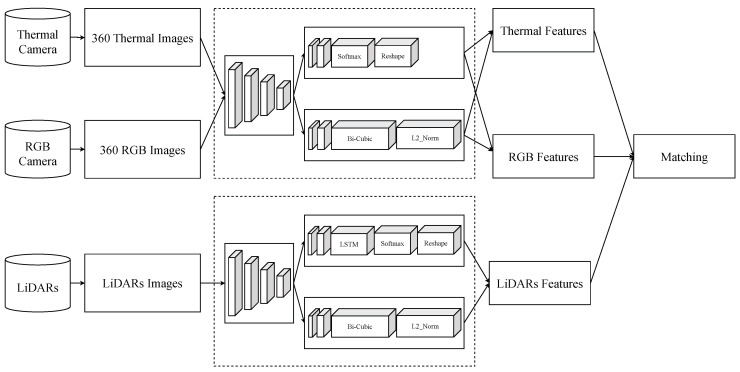
Visualization of architecture for feature extraction by the Superpoint algorithm. The system begins with data acquisition from three sensors: thermal cameras, RGB cameras, and LiDAR units. Panoramic 360 thermal images are generated by stitching frames from six thermal cameras, while five RGB cameras are used to construct a 360 RGB view. The Superpoint model for LiDAR sensors added a long short-term memory cell.

**Figure 8:**
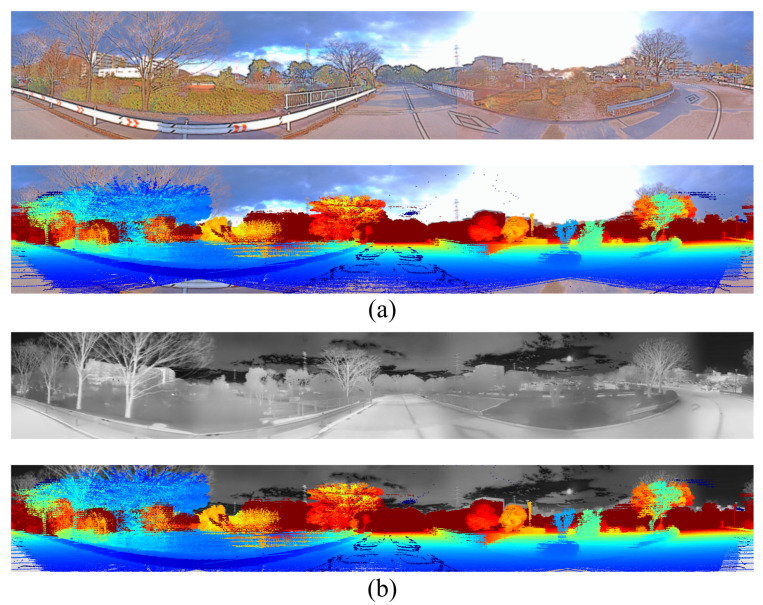
Visualization of LiDAR sensor–camera calibration; 360 RGB thermal images’ calibration. (**a**) The result of LiDAR sensor–RGB camera. (**b**) The result of LiDAR sensor–thermal camera.

Our calibration pipeline begins with the extraction of salient features from both 2D images and 3D point clouds using the SuperPoint detector [38], which provides robust and repeatable key points across different modalities, as in Figure 7. The features of the LiDAR images, 360 RGB images, and 360 thermal images are matched by the Euclidean distance, as in Equation (6), and the histogram of oriented gradients, as in Equation (7). RGB images are enhanced by Retinex decomposition to enhance fine details in poorly lit areas without compromising natural colors [39]. Outliers are then filtered using the RANSAC algorithm [40] with the Mahalanobis distance [41], as in Equation (8). *Q* is the set of points, $\vec{x}$ is the vector of points, $\vec{\mu }$ is the mean vector of the set, and *S* is the covariance matrix of the set. The calibration results are shown in Figure 8.

(6) $$\sqrt{{\left(v_{ax}-l_{bx}\right)}^{2}+{\left(v_{ay}-l_{by}\right)}^{2}}\leq \Delta \left(x,y\right)$$

(7) $$arctan\left(\frac{G_{vy}}{G_{vx}}\right)-arctan\left(\frac{G_{iy}}{G_{ix}}\right)\leq \Delta \theta$$

(8) $$d_{M}\left(\vec{x},Q\right)=\sqrt{{\left(\vec{x}-\vec{\mu }\right)}^{T}S^{-1}\left(\vec{x}-\vec{\mu }\right)}$$

## 4. Object Detection

Integrating data from multiple sensors are essential to enhance both the efficiency and robustness of object detection systems. Each sensor contributes a unique characteristic, and their combination enables a more comprehensive understanding of the environment. This fusion improves detection accuracy in complex environments with varying illumination, occlusions, or adverse weather conditions. It also improves the ability to distinguish between objects with similar shapes but different thermal or visual characteristics, improving reliability in safety-critical applications.

### 4.1. Point Cloud Accumulation

**Figure 9:**
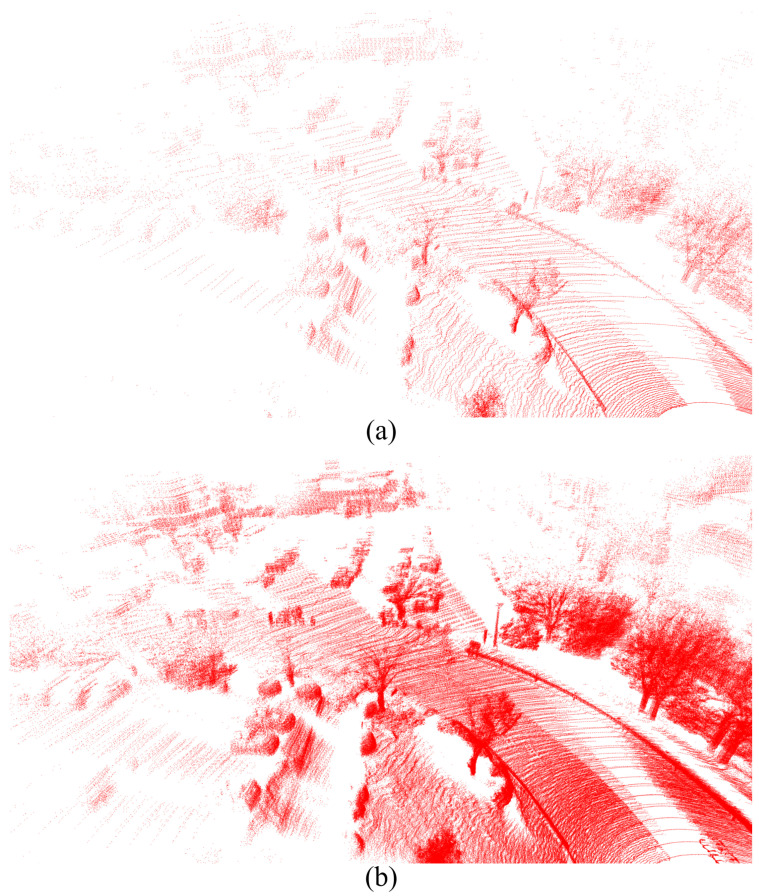
Visualization of accumulation points. (**a**) Accumulation points of four LiDAR sensors in one frame. (**b**) Accumulation points of four LiDAR sensors in seven frames.

We enhance point cloud density by aggregating data across *n* consecutive frames as in Figure 9. This temporal accumulation not only enhances the overall density of points and continuity of surfaces but also provides significant benefits when combining LiDAR with RGB and thermal images. Specifically, dense point clouds minimize alignment errors due to sparsity or incomplete surface scanning. Such improvements are particularly valuable in feature-level fusion architectures, where the integrity and precision of the input data are critical to achieve high-performance object detection and recognition. To ensure geometric consistency during temporal accumulation when combining data from multiple LiDAR sensors with potentially different positions, orientations, and scanning patterns, we apply the same enhanced ICP-based calibration algorithm described above for multi-LiDAR sensor calibration. This robust registration approach suppresses outlier influence and maintains structural coherence and reduces artifacts caused by motion or sensor misalignment. The result is temporally and spatially calibrated point clouds that serves as a high-fidelity geometric foundation for multimodal fusion with RGB and thermal imagery.

The number of *n* frames depends on the point density efficiency and camera quality. Increasing the number of points from many frames if exceeding the observation capacity of RGB cameras and thermal cameras will not be effective for observation. We use the Pixels Per Meter (PPM) or Pixels Per Foot (PPF) algorithm for object recognition according to Equation (9). *f* is the frequency of LiDAR, *v* is the speed of vehicle or LiDAR, *D* is the distance of the object, *h* is the height of the object, *P* is the resolution of the camera, *p* is the Pixel Per Meter of the object, and θ is the field of view.

(9) $$D=\frac{hR}{2p\tan(\frac{\theta }{2})}\;\mathrm{and}\;D=v\frac{n}{f}\;\Rightarrow \;n=\frac{hR}{2p\tan(\frac{\theta }{2})}\frac{f}{v}$$

### 4.2. Annotation

To label the point cloud data, we use the Latte tool [42] and the OpenPCDet open source toolkit [43], which are reliable frameworks for the detection and annotation of 3D objects. Point clouds from multiple consecutive LiDAR frames are accumulated to increase the spatial density and continuity of point cloud data and minimize the chance of missing critical surface details due to occlusions, low reflectivity, or the limited resolution of individual frames, making the labeling process both faster and more accurate.

#### 4.2.1. Data Collection

In this stage, we adopt the same accumulation strategy as described in the point clouds’ accumulation section. The primary distinction between applying accumulation for labeling and for inference lies in the number of frames used in each context. For object detection, the *n* accumulated frames must be chosen to ensure that the maximum distance gap still allows for the reliable identification of objects on RGB and thermal images. If the number of frames exceeds *n*, objects located at greater distances may fall outside the visible range of the RGB or thermal sensors, resulting in detection that relies exclusively on LiDAR data and potentially compromising the advantages of multimodal fusion.

In contrast, during the labeling process, the *t* frames used for accumulation must be configured to avoid the duplication of frames in consecutive accumulation groups. Such frame duplication may lead to inconsistent annotations and introduce noise during training. Maintaining strict non-overlapping frame windows during the labeling phase helps preserve annotation integrity and ensures that the accumulated point cloud data remain consistent and unambiguous throughout the training process.

#### 4.2.2. Annotation

**Figure 10:**
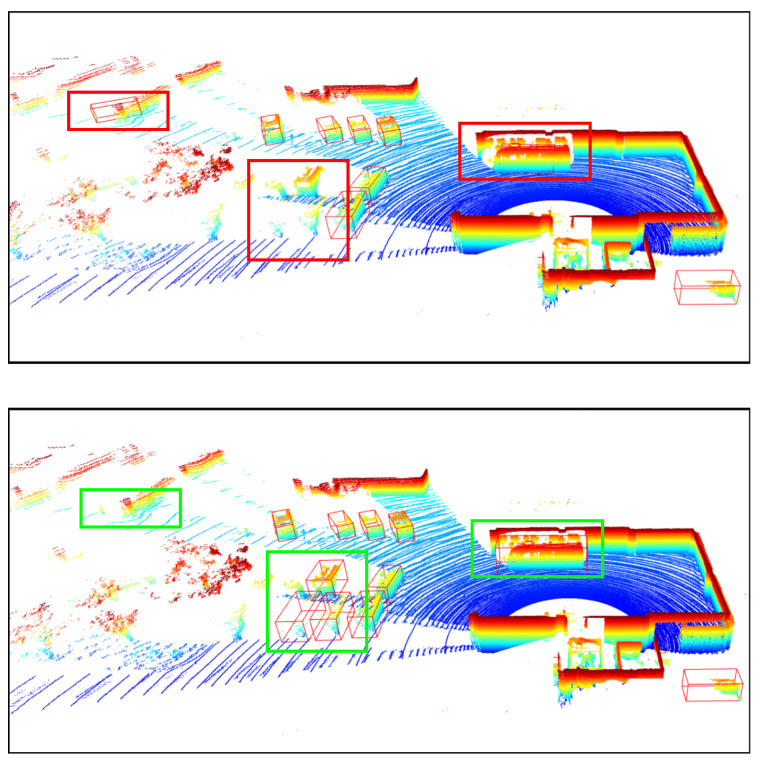
The **top image** shows bounding boxes labeled by the ReID module, with the red rectangles indicating erroneous cases. The **bottom image** shows the bounding boxes after being processed by Latte, with the green rectangles indicating refined errors.

In this stage, we perform a two-step process for automatic labeling. The first step involves coarse labeling through ReID based on an offline tracking framework. The system begins by utilizing the initial tracking results generated by an offline tracker, where object trajectories may be fragmented due to temporary occlusions. Each terminated tracklet is treated as a historical segment, while each newly appearing tracklet is considered a future candidate. The ReID module evaluates all potential history–future tracklet pairs [44]. Compared to conventional methods, this approach leverages temporal information across variable intervals, leading to more consistent labeling and improved overall annotation quality. The second step involves fine-grained refinement using the Latte toolkit, which focuses on correcting incorrect or missing boundaries, as shown in Figure 10. By combining frames, the method enhances annotation quality and reduces the manual effort and time required to label new datasets, enabling scalable annotation.

### 4.3. Network for Fusion

**Figure 11:**
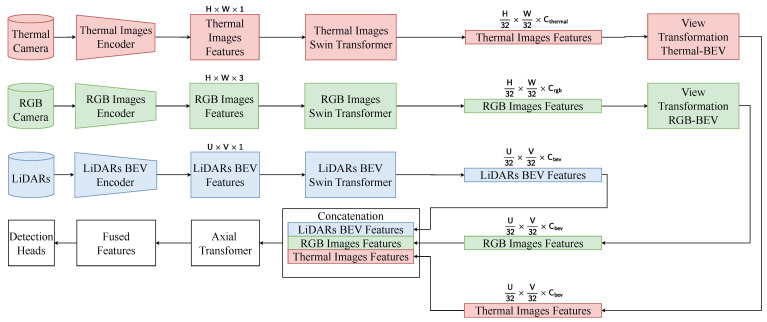
General architecture of the entire system. Features of thermal images, RGB images, and point clouds are extracted by three encoders. Then, these features are processed by Swin Transformers to reduce spatial resolution while increasing feature abstraction. These features from RGB images and thermal images are transformed before concatenated with LiDAR features. This concatenation is fused by Axial Transformer and used for object detection.

The overall architecture is shown in Figure 11. The architecture consists of three parallel branches corresponding to the RGB camera, thermal camera, and LiDAR sensors. Each branch is designed to extract features before spatially aligning them in a unified bird’s eye view (BEV) space and performing modal fusion for 3D object detection. The RGB and thermal images, with an original size of H×W×3 and H×W×1, respectively, are first passed through dedicated encoders to extract low-level modality features. These are then processed by separate Swin Transformers [45] to capture local and global dependencies within each modality. The output features of the Swin Transformers have spatial dimensions reduced to $\frac{H}{32}\times \frac{W}{32}\times C_{\mathrm{rgb}}$ and $\frac{H}{32}\times \frac{W}{32}\times C_{\mathrm{thermal}}$, respectively. Subsequently, a view transformation module is applied to both feature maps to project them into the BEV space, ensuring spatial alignment with the LiDAR domain. The raw LiDAR point clouds are encoded in BEV features of shape U×V×1, using a LiDAR BEV encoder. These features are further refined by a BEV-specific Swin Transformer, resulting in a shape output $\frac{U}{32}\times \frac{V}{32}\times C_{\mathrm{bev}}$.

The BEV-aligned features of the three modalities are then concatenated along the channel dimension and passed through an Axial Attention Transformer module [46,47], which applies axis-wise attention to capture long-range dependencies both within and between modalities. This fusion module enhances the interaction between heterogeneous features while maintaining the critical spatial layout for 3D understanding. Finally, the fused BEV features are forwarded to task-specific detection heads to generate the final 3D object predictions.

#### 4.3.1. Feature Encoder

**Figure 12:**
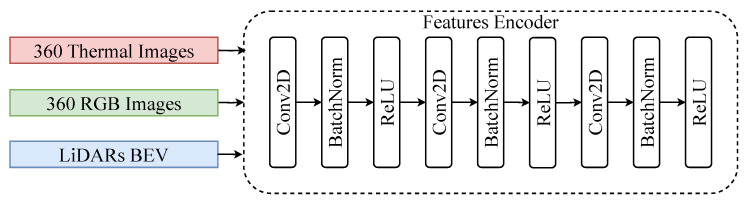
Visualization of encoder blocks; 360 RGB images, 360 thermal images, and LiDAR sensor point clouds are applied to these blocks for feature extraction.

To extract features from the RGB images, thermal images, and LiDAR BEV representations, a modality-specific but structurally unified encoder architecture is employed. Each encoder is composed of three consecutive convolutional blocks, where each block contains a 2D convolutional layer followed by a batch normalization layer and a ReLU activation function [48,49], as illustrated in Figure 12. These blocks operate on the spatial dimensions of the input tensor, with the convolutional layers designed to capture local contextual information, such as edges, textures, and shape boundaries. The depth and stride of the convolutional layers are selected to progressively reduce resolution. Batch normalization is applied to stabilize the training in the presence of heterogeneous input distributions. The inclusion of ReLU nonlinearity promotes feature disentanglement and improves convergence. This design facilitates the projection of all modalities into a shared feature space, thus simplifying multimodal fusion while maintaining sensitivity to modality-specific characteristics.

#### 4.3.2. Swin Transformer

**Figure 13:**
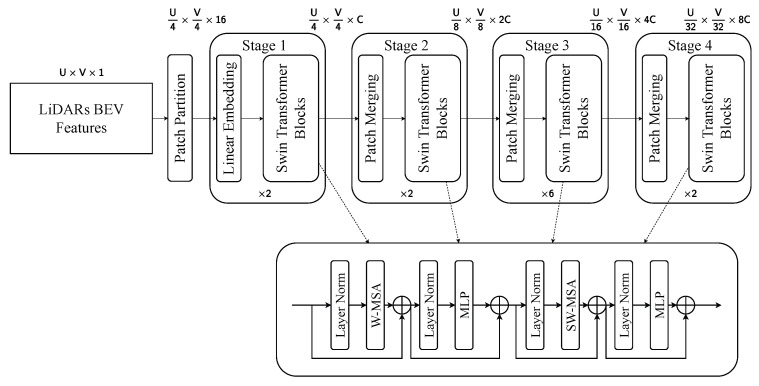
Visualization of Swin Transformer for LiDAR features. This pipeline extracts intensity information from point clouds.

**Figure 14:**
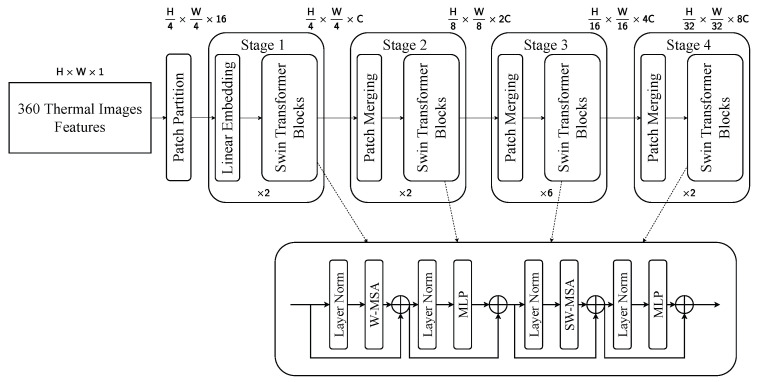
Visualization of Swin Transformer for thermal image features. This pipeline extracts thermal information from thermal images.

The thermal and LiDAR BEV branches utilize a hierarchical Swin Transformer architecture composed of four stages, as depicted in Figure 13 and Figure 14. The input features, originally sized H×W×1 for thermal images and U×V×1 for the BEV projection, are first partitioned into 4×4 patches. Each Swin Transformer block contains a multihead window self-attention module (W-MSA) and a shifted window attention module (SW-MSA) [45], alternating to allow local and inter-region interactions. These attention mechanisms are encapsulated between layer normalization and a multilayer perceptron, forming a residual connection structure to facilitate gradient propagation and feature refinement. This architecture effectively captures modality-specific features.

**Figure 15:**
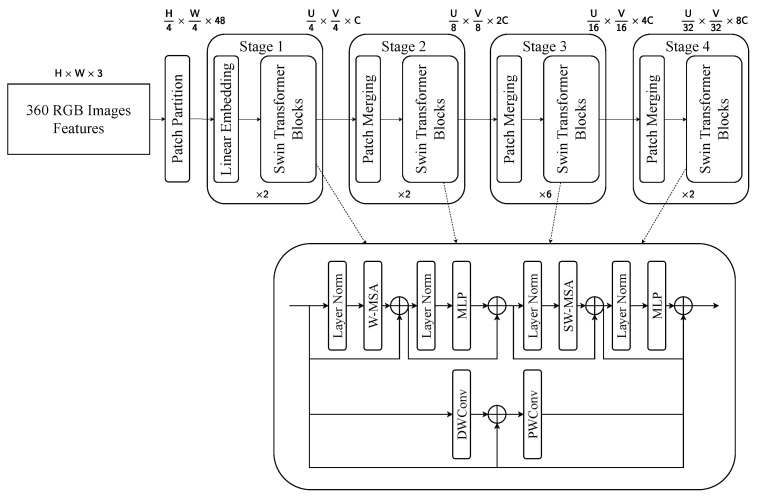
Visualization of Swin Transformer for RGB image features. This pipeline extracts the three color channels from RGB images.

The RGB branch adopts the same hierarchical structure, but introduces a modified feedforward module within each Swin Transformer block to better exploit the rich spatial and spectral content of RGB images, as shown in Figure 15. The input, with a shape of H×W×3, is divided into 4×4 patches and projected into an initial embedding of size $\frac{H}{4}\times \frac{W}{4}\times 48$. The larger channel dimension in the embedding stage is responsible for the higher information density present in RGB inputs. Stage 4 outputs the final representations of the dimension $\frac{H}{32}\times \frac{W}{32}\times 8C$ through two remaining blocks. Unlike the thermal and BEV branches, the RGB variant uses a dual-branch convolutional unit consisting of depth-wise and point-wise convolution [50,51] to enhance the ability to learn localized texture and edge information.

#### 4.3.3. View Transformation

The purpose of Algorithm 1 is to transform the features of the 2D image from a perspective view into a spatially aligned representation in the BEV space. Each pixel is associated with a horizontal angle or yaw angle and a vertical angle or pitch angle. The image feature is then projected into a BEV feature vector. If multiple image pixels project to the same BEV cell, features are aggregated using a strategy such as summation, mean, or max pooling. This allows spatially overlapping rays to contribute information to the same location in BEV space. In general, this view transformation aligns perspective view features into a top-down geometric structure without relying on depth sensors or learned depth prediction. It serves as a bridge between image-space representation and map-like spatial reasoning, enabling later stages to operate on spatially consistent features across modalities.

**Algorithm 1** View Transformation 2D-BEV    INPUT
         $2D\;\mathrm{Feature}\;\mathrm{map}:\;F\in R^{H\times W\times C_{2}D}$    PARAMETERS        $\mathrm{Vertical}\mathrm{field}\mathrm{of}\mathrm{view}:\phi_{up},\phi_{down}$        SizeofBEV:U×V         $\mathrm{Origin}\mathrm{of}\mathrm{the}\mathrm{BEV}\mathrm{grid}:x_{0},y_{0}$        Gridresolution:r∈R        Linearprojectionweightsandbias:w,b        Sensorheight:h    OUTPUT        $\mathrm{BEV}\mathrm{Feature}\mathrm{map}:B\in R^{U\times V\times C_{\mathrm{bev}}}$    RESULTS        fori=0toH−1             forj=0toW−1             $\psi \left(yaw\right)\leftarrow \pi \left(2\frac{j}{W}-1\right)$             $\theta \left(pitch\right)\leftarrow \phi_{down}+\frac{i}{H}\left(\phi_{up}-\phi_{down}\right)$             $d\leftarrow \left[\begin{array}{c} \cos\theta \cos\psi \\ -\cos\theta \sin\psi \\ \sin\theta \end{array}\right]\in R^{3}$             ifsinθ≠0                  $t\leftarrow \frac{h}{\sin\theta }$                  (x,y)←(tcosθcosψ,−tcosθsinψ)                  $u\leftarrow \lfloor \frac{x-x_{0}}{r}\rfloor$                  $v\leftarrow \lfloor \frac{y-y_{0}}{r}\rfloor$                  if 0≤u<U and 0≤v<V                       $f_{\mathrm{bev}}\leftarrow wF\left[i,j,:\right]+b$                       $B\left[u,v,c\right]\leftarrow aggregate(B\left[u,v,c\right],f_{\mathrm{bev}}\left[c\right])$

#### 4.3.4. Axial Transformer

**Figure 16:**
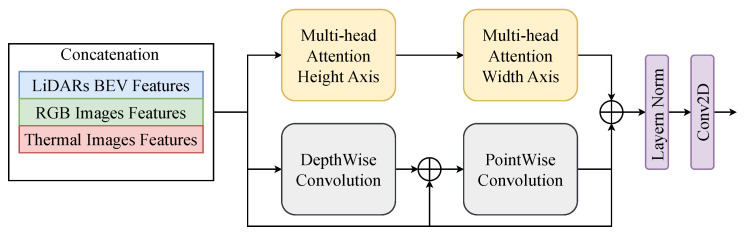
Visualization of Axial Transformer for fusing multimodal features. Concatenated features are applied in a parallel structure. Then, the output is re-adjusted by the Layer Norm and Conv2D.

To integrate heterogeneous features extracted from the LiDAR BEV, RGB, and thermal modalities, we propose a dedicated fusion module, as in Figure 16. This module is applied after modality-specific Swin Transformer modules, where the features of all three branches are spatially aligned and concatenated, resulting in a unified representation of size $\frac{U}{32}\;\times \;\frac{V}{32}\;\times \;C_{\mathrm{fused}}$. The fusion process begins with two consecutive multihead self-attention operations [46,47]. The first applies attention along the height axis and the second applies attention along the width axis. Using two-axis modules reduces computational complexity while maintaining the ability to learn structured spatial relationships between features from different modalities. In parallel with the attention pathway, the fused representation is also processed by a depth-wise convolution followed by a point-wise convolution [51]. The outputs of the axis-attention branch and the convolutional branch are summed and passed through a residual connection, then normalized and refined by a final 2D convolution layer, producing the fused representation. Axis-wise attention facilitates alignment across modalities in both spatial dimensions, while the depth-wise and point-wise convolution paths ensure that local structural cues are preserved. As a result, the fusion module generates rich and coherent features that improve robustness in multimodal 3D object detection tasks. In the last stage, the Layer Norm normalizes the input to help achieve stable convergence, while the Conv2D helps encode local features and re-adjust geometric information.

The detection network is trained using a composite loss function that jointly supervises object classification and 3D bounding box regression. The total loss is defined as Equation (10).

(10) $$L=\lambda_{1}L_{1}+\lambda_{2}L_{2}$$

where $L_{1}$ is the classification loss and $L_{2}$ is the bounding box loss. The classification loss is calculated using the cross-entropy loss function in Equation (11). $\hat{q}=\left[{\hat{q}}_{1},{\hat{q}}_{2},\ldots ,\hat{q_{K}}\right]$ is a probability distribution in K object classes. The ground truth label is encoded as a one-hot vector $p=[p_{1},p_{2},\ldots ,p_{K}]$

(11) $$L_{1}=-\sum_{k=1}^{K}p_{k}\log\left(\hat{q_{k}}\right)$$

The bounding box loss is calculated using the Huber loss function, as in Equation (12). $b_{j}\;=\;\left\{x_{j},y_{j},z_{j},h_{j},w_{j},l_{j},\theta_{j}\right\}$ represents the 3D bounding box parameters and $b\;=\;\{b_{1},b_{2},\ldots ,b_{N}\}$ represents the set of 3D bounding box parameters for *N* objects.

(12) $$L_{2}=\sum_{n=1}^{N}L_{\delta }\Delta b_{n}=\sum_{n=1}^{N}L_{\delta }\left(b_{n}-{\hat{b}}_{n}\right)\;\mathrm{where}\;L_{\delta }\Delta b=\left\{\begin{array}{c} \frac{1}{2}b^{2},\;\mathrm{if}\;\left|\Delta b\right|\leq \delta \\ \delta (|\Delta b|-\frac{1}{2}\delta ),\;\mathrm{otherwise} \end{array}\right.$$

## 5. Experiment Results

To evaluate the effectiveness of our proposed method, we performed experiments on a comprehensive dataset comprising synchronized RGB images, thermal images, and LiDAR point clouds. The RGB imagery was captured using five out of six cameras from the Ladybug5 system, each with a horizontal field of view (FoV) of 90 degrees, and thermal images were acquired from six FLIR ADK cameras, each offering a 75-degree FoV. The two camera systems were arranged in a circular configuration to approximate panoramic thermal coverage.

The LiDAR data were collected from Ouster 128, Ouster 64, Velodyne Alpha Prime, and Hesai Pandar. The Ouster OS1-128 and OS1-64 devices have vertical FoVs ranging from −22.5 to +22.5 degrees, with 128 and 64 laser channels, respectively. The Velodyne Alpha Prime is configured with 128 channels and a vertical FoV ranging from −15 to +15 degrees, making it suitable for long-range high-resolution sensing. Meanwhile, Hesai Pandar64 provides 64 laser channels and a vertical FoV of approximately −16.6 to +16.6 degrees, offering balanced performance for mid-range perception. In all the evaluation sections, we only evaluated the accuracy of car detection.

The input data consist of LiDAR point clouds with 3,487,243 points per frame, 360 RGB images with a resolution of 2314×400, and 360 thermal images with a resolution of 2365×340. In the model, the Adam optimizer uses a learning rate of 0.0001, a momentum of 0.9, and a weight decay of 0.01. The model is trained for 80 epochs with a batch size of 16. This study was conducted with an RTX 3050 GPU.

### 5.1. Comparison with State-of-the-Art Registration Methods

We first assessed the precision of our calibration procedure by comparing it with several calibration methods. These methods are applied in LiDAR camera calibration and registration. For comparison, all methods were initialized with the same sensor configuration. To quantitatively assess alignment quality, we used two metrics: Root Mean Square Error [52] and Recall [53].

1. Root Mean Square Error (RMSE) measures the average Euclidean distance between the corresponding pair of points after registration. Lower RMSE values correspond to more accurate and reliable transformations. It serves as an indicator of how well the transformed source point clouds align with the target. In our implementation, these correspondences are not manually annotated, but are established automatically during the registration process. Specifically, the algorithm performs iterative nearest-neighbor matching to determine point-to-point correspondences, which are then refined throughout the optimization. Only inliers, points within a predefined distance threshold, are used in the RMSE computation to ensure that the metric reflects a meaningful alignment accuracy. A lower RMSE indicates a more precise and reliable transformation.
  (13) $$RMSE=\sqrt{\frac{1}{N}\sum_{i=1}^{N}{\left(q_{i}-q_{i}^{T}\right)}^{2}}$$
2. Recall measures the ability of the detector to find all relevant objects in the image. High recall means that the model detects most of the actual objects, even if some detections are inaccurate or redundant. A True Positive (TP) result indicates a correct detection and a False Negative (FN) result indicates a ground truth object that is not detected.
  (14) $$Recall=\frac{\left(TP\right)}{\left(TP)+(FN\right)}$$

Table 1 presents the registration performance of various methods in a dataset consisting of 100 frame pairs collected under normal weather conditions. The results show that our proposed method achieves the highest performance, with a Recall of 97.36% and the lowest RMSE at 0.36, indicating a highly accurate and consistent alignment. Compared to classical and modern methods, our method outperforms all others in both accuracy and geometric alignment quality. Overall, the results confirm that not only is our model theoretically robust, but it also delivers superior practical performance in 3D registration tasks under standard environmental conditions.

### 5.2. Comparison with State-of-the-Art Detectors

To thoroughly and rigorously evaluate the effectiveness and robustness of our proposed system, we conducted comprehensive comparisons with several state-of-the-art approaches that have been widely applied in object detection research. The methods selected for this comparison reflect their prominence in current research and their practical applicability in single- and multimodal sensing systems. All evaluated methods and our proposed approach are consistently trained, validated, and tested using the same experimental setup, dataset, and sensor configurations to ensure the fairness and integrity of comparative analysis. Furthermore, we consider that a considerable portion of previous research has focused primarily on evaluating detection algorithms under optimal environmental conditions with clear visibility and minimal disturbances. Such an idealized scenario may not sufficiently represent the challenges encountered in practical deployments. To address this limitation in a comprehensive way, we expand our evaluation approach into two distinct phases to fully reflect realistic conditions.

In the first evaluation, we performed experiments using datasets collected under normal environmental conditions characterized by clear weather and ideal lighting. This dataset represents a scenario in which most multimodal object detection algorithms typically demonstrate high performance and reliability. In contrast, the second and third evaluations involve rigorous testing using datasets acquired under adverse environmental conditions, including scenarios with reflective surfaces, thermal interference, and poor visibility due to weather phenomena such as snow or night. These challenging circumstances significantly impact sensor effectiveness and pose notable difficulties for detection algorithms. By implementing this two-comprehensive evaluation framework, our aim is to systematically assess and highlight the performance, strengths, and limitations of each fusion method in varying operational scenarios. To quantify and objectively analyze detection results, we utilize standard performance metrics, including Intersection over Union and Average Precision, thereby ensuring that our findings are both reliable and widely comparable within the research community:

1. Intersection over Union (IoU) [59] is a measure of the overlap between the predicted bounding box and the ground truth bounding box. A detection is considered correct if the IoU between the predicted and ground truth box exceeds a predefined threshold. The area of overlap is the shared area between the predicted box and the ground truth box. The area of union is the total area covered by both boxes.
  (15) $$IoU=\frac{Area\;of\;Overlap}{Area\;of\;Union}$$
2. The Average Precision (AP) [53] in Equations (14), (16) and (17) is a measure of the general accuracy of the model in detecting objects, calculated based on the Precision–Recall curve at various prediction thresholds. The higher and more stable the prediction, the larger the AP. There are two types of AP commonly used in 3D object detection problems: $AP_{BEV}$ and $AP_{3D}$. $AP_{BEV}$ compares the predicted box and the ground truth box in terms of location and area in a two-dimensional plane. $AP_{3D}$ compares the predicted box and the ground truth box in terms of location and volume in a three-dimensional space. A False Positive (FP) result indicates a predicted object that does not correspond to any ground truth object.
  (16) $$AP=\sum_{i=1}^{N}\left(R_{i}-R_{i-1}\right)P_{i}\;\mathrm{where}\;R=Recall;P=Precision$$
  (17) $$Precision=\frac{\left(TP\right)}{\left(TP)+(FP\right)}$$

We will evaluate and compare the algorithm’s performance across three distinct environmental conditions. The first scenario corresponds to typical conditions without significant environmental interference. The second scenario involves data collection in a snowy environment. The final scenario is set at night. Under normal conditions, both LiDAR sensors and cameras operate optimally, facilitating accurate and straightforward object detection using any type of sensor. In the snowy environment, LiDAR sensors and thermal cameras become more susceptible to interference from their surroundings, whereas RGB cameras maintain relatively stable performance. In contrast, at night, RGB cameras experience significant performance degradation compared to LiDAR and thermal cameras. Conducting experiments across multiple environmental scenarios allows us to thoroughly evaluate and validate the robustness and effectiveness of our proposed method. We evaluated the performance with IoU as 0.5.

#### 5.2.1. Evaluation Under Normal Conditions

**Figure 17:**
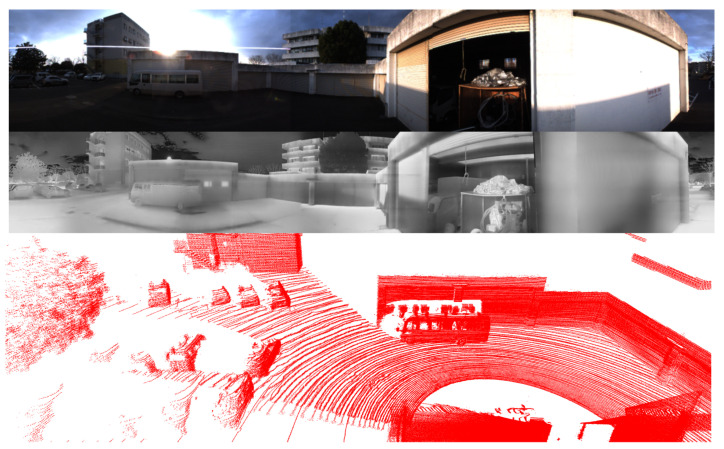
Visualization of all sensors under normal conditions. RGB images, thermal images, and point clouds capture clear data.

Table 2 presents a comparative analysis of several methods under normal environmental conditions. In this scenario, sensors operate optimally without interference from weather conditions or variations due to time of day. An illustrative example is provided in Figure 17, which highlights the clarity and high fidelity of the captured data. In such settings, sensors are able to perform at their full potential, without being impeded by external environmental factors. Any noise or point cloud distortions predominantly originate from the inherent properties of LiDAR systems when they interact with specific materials or object geometries, rather than being attributable to external environmental factors.

#### 5.2.2. Evaluation Under Snowy Daytime Conditions

**Figure 18:**
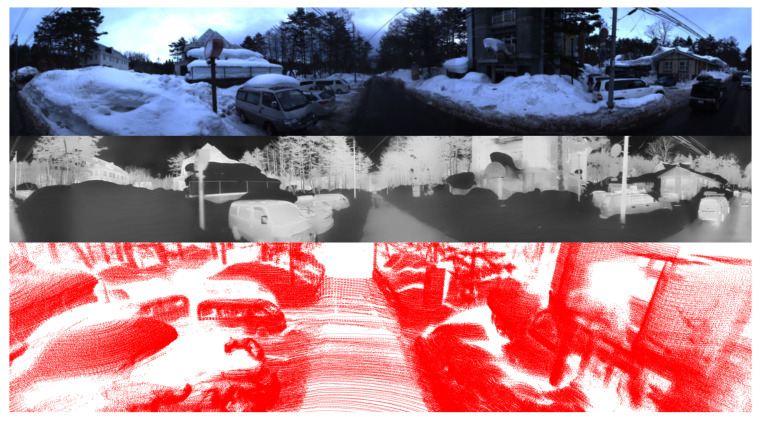
Visualization of LiDAR point clouds and thermal images affected by snowy daytime conditions. RGB images play a more significant role.

In Table 3, we present a comparative evaluation of various methods under environmental conditions characterized by the presence of snow. In this experiment, data acquisition was carried out during periods of the day, enabling RGB cameras to collect high-quality imagery, as exemplified in Figure 18. However, the snowy environment introduced significant interference to both LiDAR sensors and thermal cameras. Noise points are generated by substantial laser light reflection from snow and ice. This phenomenon significantly diminishes the quality of point cloud data, thus impairing the precision and accuracy of object detection tasks utilizing LiDAR sensors. In addition, the cold environment associated with snow induces thermal uniformity, thereby reducing the thermal contrast between objects and surroundings. Among the sensor modalities evaluated, RGB cameras generally experience the least degradation in performance, as they preserve essential visual attributes such as color, shape, and texture. Although the predominantly white background created by snow may reduce contrast, it typically does not lead to significant deterioration in detection performance, particularly compared to the challenges encountered by LiDAR.

The results shown in Table 3 clearly illustrate the advantage of incorporating RGB cameras compared to LiDAR-only approaches. LiDAR-only methods experience substantial performance declines under snowy conditions due to environmental interference. In contrast, methods that employ a combination of LiDAR and RGB cameras exhibit stability in both BEV and 3D object detection. Generally, in the snowy conditions evaluated here, the integration of RGB cameras emerges as the most impactful strategy to improve the overall 3D object detection reliability and accuracy. Compared with existing approaches for one frame, our proposed method demonstrates noticeable improvements in detection performance under snow conditions. Moreover, the presence of outliers is significantly reduced across varying distances due to the comprehensive spatial coverage provided by the four LiDAR sensors at close, medium, and long ranges.

#### 5.2.3. Evaluation Under Snowy Nighttime Conditions

**Figure 19:**
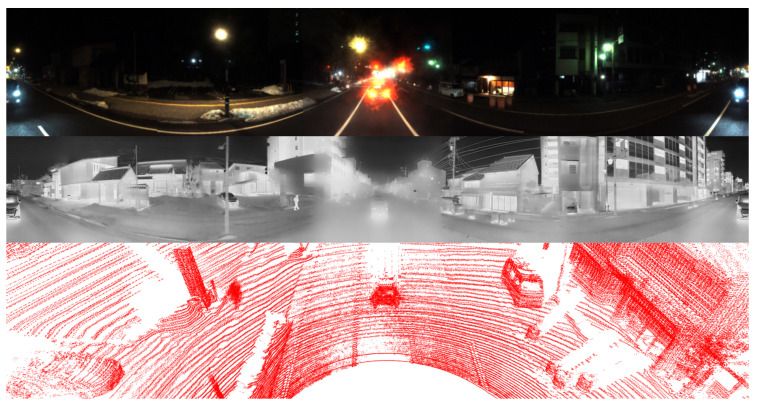
Visualization of RGB images affected by snowy nighttime conditions. LiDAR and thermal images play a more significant role.

In this evaluation, we compare the performance of various methods on a snowy night with that in Table 4. In such environments, the quality of images captured by RGB cameras deteriorates significantly due to the absence of natural illumination, even when image enhancement tools such as Retinex are used, as shown in Figure 19. As a result, the detection performance that relies on RGB data becomes unreliable. Under these challenging lighting conditions, LiDAR sensors and thermal cameras demonstrate clear advantages. LiDAR sensors operate independently of ambient light and thus remain stable and reliable at night. The absence of sunlight minimizes background interference, leading to cleaner point clouds with reduced noise levels.

Thermal cameras also perform more effectively at night, as thermal contrast between objects and the surrounding environment tends to be greater in the absence of solar radiation. This increased contrast improves the clarity and distinctiveness of thermal signatures, allowing thermal cameras to detect objects with greater precision. Although cold temperatures, especially during winter, can reduce the thermal difference between objects and the environment, thermal imaging still generally outperforms RGB imaging under these conditions. Although RGB cameras may still be usable at night when supported by artificial lighting, their performance remains inconsistent and prone to errors. In contrast, fusion-based approaches that integrate LiDAR with thermal imaging exhibit stronger robustness and reliability. The performance difference between systems with and without RGB cameras becomes less significant at night.

Similarly to the above situations, the use of multiple LiDAR sensors offers substantial benefits by increasing point cloud density and further reducing spatial noise. In low light conditions, where sunlight interference is absent, all four LiDAR sensors tend to operate more stably, producing cleaner and more consistent point cloud data. The denser and more reliable point distribution improves the system’s capacity for accurate spatial perception. The experimental results clearly demonstrate that our approach, when supported by multiple LiDAR sensors, outperforms several existing methods.

### 5.3. Ablation Studies

In the previous evaluation, we presented a comprehensive evaluation by comparing our proposed method with several existing approaches under different conditions. Although the comparison with other methods provided a broad overview of performance, the ablation studies aimed to conduct a more focused and detailed analysis to further demonstrate the effectiveness and robustness of our method.

#### 5.3.1. Evaluation of Effectiveness of LiDAR Sensors at Different Distances

To further understand the performance and limitations of different LiDAR configurations, we performed a distance-based evaluation of 3D object detection. In real-world autonomous systems, objects appear in varying ranges from the sensor, and the effectiveness of a LiDAR can vary significantly with distance because of differences in resolution, point density, and sensor placement. Therefore, it is essential to assess whether sensor fusion strategies can maintain detection quality in the near, mid, and far ranges. Table 5 presents the 3D Average Precision measured in distance intervals.

The configuration using four LiDAR sensors consistently achieves the best performance across all distance intervals. This confirms that multisensor fusion can effectively combine the complementary strengths of each individual sensor while reducing their limitations, offering broader spatial coverage and more robust environmental representation. Generally, instead of relying on a single sensor, combining four LiDAR sensors demonstrates clear advantages by improving detection performance across all spatial ranges.

#### 5.3.2. Evaluation of Effectiveness of Modalities

To examine the contribution of individual sensing modalities under adverse conditions, we evaluated 3D detection performance using different combinations of LiDAR, RGB camera, and thermal camera. Specifically, we consider scenarios under snowfall and nighttime, where perception becomes particularly challenging due to reduced visibility. Table 6 presents the Average Precision under these conditions for various configurations of modality.

The results indicate that using only LiDAR, or selectively disabling either the RGB or thermal branch, still produces acceptable detection outputs depending on situations. Each modality, on its own or in partial combinations, is capable of contributing meaningful cues depending on the situation. However, the integration of LiDAR, RGB, and thermal data provides a more complete understanding of the scene, enhancing stability. These findings underscore the importance of multimodal fusion in real-world applications where environmental variability is inevitable.

#### 5.3.3. Evaluation of Effectiveness of Each Module

To evaluate the individual contribution of each module in our architecture, we conducted an ablation study by systematically replacing key components with simpler alternatives. Specifically, the focal loss is replaced with the standard cross-entropy loss, the Swin Transformer is replaced with average pooling, and the axial attention fusion module is replaced with a simple feature concatenation strategy. We also evaluated the effect of removing the depthwise separable convolution, which is designed to improve efficiency in the fusion process.

As shown in Table 7, each simplified configuration leads to a decrease in detection performance, indicating that the original design of each module plays a significant role in the final result. Simpler alternatives may offer speed, but combining all modules yields the highest detection accuracy.

### 5.4. Qualitative Results

**Figure 20:**
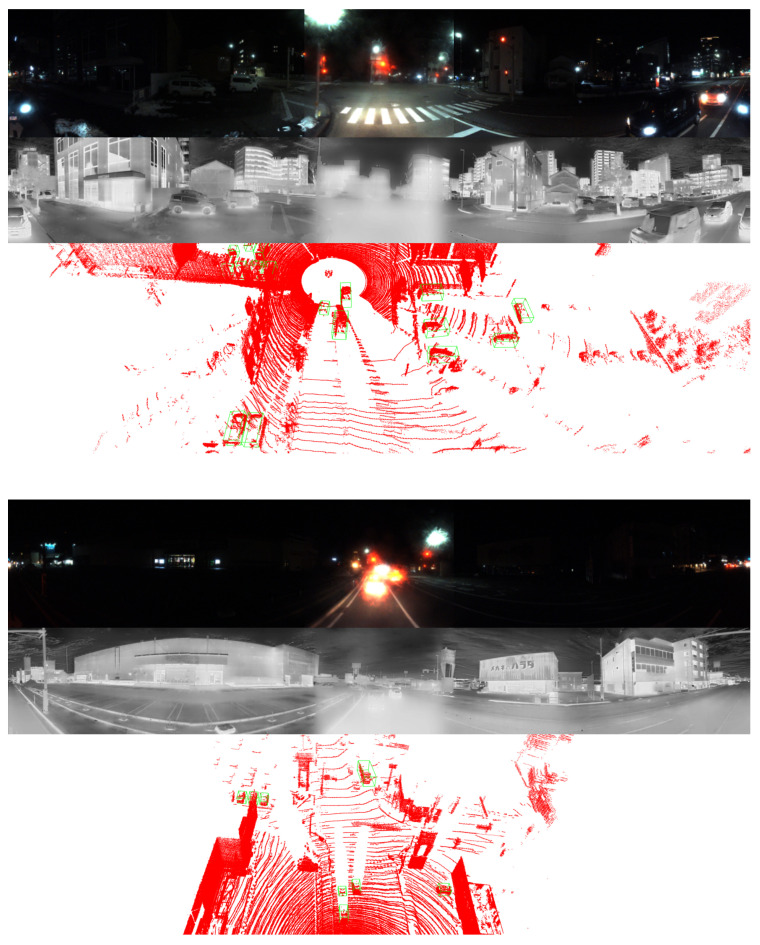
Visualization of predicted bounding boxes under snowy nighttime conditions.

**Figure 21:**
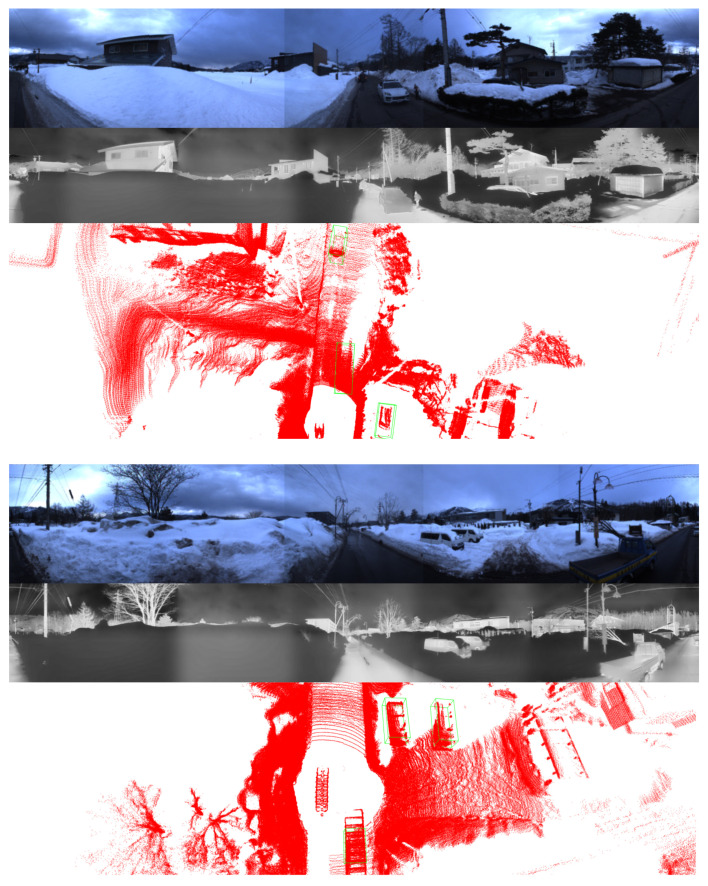
Visualization of predicted bounding boxes under snowy daytime conditions.

In this section, we present the qualitative results of our proposed model under particularly challenging conditions, as shown in Figure 20. RGB images are significantly degraded due to insufficient illumination or strong light interference, which limits their effectiveness. In contrast, in snowy daytime conditions as in Figure 21, LiDAR sensors often suffer from severe noise caused by snowflakes and reflective surfaces, while thermal cameras may experience reduced contrast due to uniformly low temperatures.

The comparison between the two scenarios underscores the effectiveness of the proposed multimodal fusion framework in addressing a wide range of visual challenges. In nighttime environments, the RGB modality is significantly degraded due to limited illumination, intense glare from artificial lights, and increased visual noise. Under such conditions, thermal imaging offers greater stability, while LiDAR gives a reliable geometric structure for accurate 3D localization. In contrast, snowy daytime conditions introduce a different set of challenges. The presence of heavy snow results in strong reflectivity, which adversely impacts the quality of LiDAR measurements. At the same time, uniformly low temperatures reduce the effectiveness of thermal imaging in distinguishing objects, except for those with notable heat emission. Despite these difficulties, the predicted 3D bounding boxes remain consistently well aligned across all modalities, demonstrating the effectiveness of the proposed approach. These results confirm that the integration of RGB, thermal, and LiDAR data enables the system to adapt to varying environmental conditions and maintain high detection accuracy, even when individual sensing modalities are compromised.

## 6. Discussion

**Figure 22:**
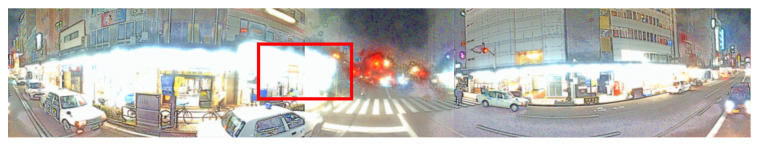
Visualization of the overlapping areas of the RGB cameras is affected by strong light.

Although many advances have been mentioned in this paper, there are still some challenges. First, although the use of 360 cameras can completely solve the problems related to blind spots, making the field of view wider, if there is a strong light source shining directly on the intersection area of the two cameras, the effect of light streaks cannot be solved by conventional balancing algorithms, as in Figure 22.

In addition, the use of multiple LiDAR sensors in multiple locations helps to eliminate some blind spots and increase the density of points. Proper LiDAR sensor placement is crucial to avoid laser interference. In addition, using different LiDAR sensors with different designs will require careful intensity normalization. In contrast, using the same LiDAR sensors avoids intensity normalization, but choosing LiDAR sensors that are similar or have very similar point distributions will cause the density of points in close and far areas to be significantly different. For example, if, instead of four LiDAR sensors with different point distributions from near to far, we use four Ouster-128 LiDAR sensors, the density of points in the near area will increase dramatically, and as a consequence, deep learning methods may overfit objects at close distances.

## 7. Conclusions

In this paper, we present a novel method for combining LiDAR sensors, RGB cameras, and thermal cameras for object detection. The system integrates the advantages of many types of sensors, thereby allowing object detection in many different environments and times. To eliminate the blind spots of traditional cameras with narrow fields of view, we use 360 cameras stitched from RGB cameras and thermal cameras, thereby improving the performance of object detection, tracking problems, and SLAM problems. To enhance the density and performance of LiDAR sensors, we combine four LiDAR sensors with different fields of view and different intensities. Then, consecutive point cloud frames are also used to clarify and enhance the point density. In addition, the use of consecutive frames also helps our system minimize the loss or image distortion caused by challenging materials and atypical colors when using LiDAR sensors. After collecting data from the sensors, we propose a new architecture to improve the performance of the object detection problem. We use an advanced architecture to improve the Swin Transformer to extract features more efficiently. We then use a new axial self-attention strategy for fusion to generate rich and coherent features that improve robustness in multimodal 3D object detection tasks. Current results demonstrate effectiveness in low light and snowy conditions, affecting RGB, thermal cameras, and LiDAR sensors differently. Although there are certain improvements in performance, there are still some areas that we can improve, as mentioned above. Future work will address more extreme weather conditions like heavy rain and snow. Furthermore, our goal is to maintain the stability and object tracking ability in extreme weather conditions. We will improve and develop the program to add this method to Autoware software [17], the biggest open source project in the world for applications related to autonomous vehicles and related sensors.

## Figures and Tables

**Table 1:** Registration performance on our dataset.

Method	Recall (%)	RMSE (m)
G-ICP	94.22	0.55
D3Feat [54]	95.45	0.45
SpinNet [55]	95.72	0.43
MAC [56]	96.43	0.39
Fast and Robust ICP [57]	96.26	0.48
CAST [58]	96.81	0.34
Ours	97.36	0.36

**Table 2:** Object detection performance on our dataset under normal conditions with IoU=0.5.

Sensor	Method	${\mathrm{AP}}_{3D}$ (%)	${\mathrm{AP}}_{\mathrm{BEV}}$ (%)
LiDAR	CT3D [60]	87.22	90.81
	SASA [61]	86.94	90.98
	TED [62]	85.83	91.42
	CPD [63]	87.95	92.45
	LISO [64]	88.64	92.88
LiDAR + RGB camera	CLOCs [7]	83.34	87.60
	EPNet [27]	85.15	88.85
	FocalsConv [5]	87.34	91.63
	SFD [8]	88.53	91.76
	GraphR-CNN [28]	86.67	90.93
	MoME [9]	91.02	93.63
	EPR [10]	90.65	94.38
	Ours	91.79	95.17

**Table 3:** Object detection performance on our dataset in snowy weather with IoU=0.5.

Sensor	Method	${\mathrm{AP}}_{3D}$ (%)	${\mathrm{AP}}_{\mathrm{BEV}}$ (%)
LiDAR	CT3D	82.26	84.15
	SASA	81.85	85.22
	TED	82.43	87.96
	CPD	84.67	88.42
	LISO	85.78	87.73
LiDAR + RGB camera	CLOCs	83.86	86.97
	EPNet	84.65	87.52
	FocalsConv	83.07	89.65
	SFD	87.23	90.05
	GraphR-CNN	87.14	89.45
	MoME	88.35	90.73
	EPR	88.92	91.25
	Ours	89.58	93.35

**Table 4:** Object detection performance on our dataset at night with IoU=0.5.

Sensor	Method	${\mathrm{AP}}_{3D}$ (%)	${\mathrm{AP}}_{\mathrm{BEV}}$ (%)
LiDAR	CT3D	88.14	91.98
	SASA	88.96	91.76
	TED	87.75	92.55
	CPD	88.87	92.45
	LISO	88.64	93.24
LiDAR + RGB camera	CLOCs	82.04	85.27
	EPNet	82.89	86.12
	FocalsConv	83.31	87.58
	SFD	85.85	88.27
	GraphR-CNN	84.29	88.32
	MoME	86.93	90.48
	EPR	87.06	89.20
	Ours	91.56	94.68

**Table 5:** The 3D Average Precision at different distances.

Sensor	0–10 m	10–20 m	20–30 m	>30 m
Hesai Pandar	92.75	85.69	75.56	44.77
Velodyne	91.84	82.24	76.82	41.15
Ouster 64	93.97	81.33	62.29	26.15
Ouster 128	94.41	87.12	67.48	26.94
Four LiDARs	94.85	88.24	77.07	45.84

**Table 6:** The Average Precision across modalities.

Sensor	Snow		Night	
	${\mathrm{AP}}_{3D}$ **(%)**	${\mathrm{AP}}_{\mathrm{BEV}}$ **(%)**	${\mathrm{AP}}_{3D}$ **(%)**	${\mathrm{AP}}_{\mathrm{BEV}}$ **(%)**
only LiDAR	87.54	88.76	92.22	95.56
LiDAR + RGB	89.23	94.17	88.73	93.97
LiDAR + Thermal	87.15	92.86	91.48	95.31
Fusion	89.58	93.35	91.56	94.68

**Table 7:** The Average Precision of each module.

Focal Loss	Swin Transformer	Axial Attention Fusion	Separable Convolution	Frames of Input	${\mathrm{AP}}_{3D}$ (%)
	√	√	√	7	87.58
√		√	√	7	85.44
√	√			7	84.75
√	√	√		7	86.20
√	√	√	√	1	82.20
√	√	√	√	7	89.75

## Data Availability

The data presented in this study are available on request from the corresponding author. The data are not publicly available due to privacy.
